# Ferroelectrics with a controlled oxygen-vacancy distribution by design

**DOI:** 10.1038/s41598-019-40717-0

**Published:** 2019-03-12

**Authors:** Yuji Noguchi, Hiroki Matsuo, Yuuki Kitanaka, Masaru Miyayama

**Affiliations:** 1 Department of Applied Chemistry, School of Engineering, The University of Tokyo, 7-3-1 Hongo, Bunkyo-Ku, Tokyo 113-856 Japan; 2 Present Address: Graduate School of Frontier Sciences, The University of Tokyo, 5-1-5 Kashiwanoha, Kashiwa-shi, Chiba 277-8563 Japan

## Abstract

Controlling and manipulating defects in materials provides an extra degree of freedom not only for enhancing physical properties but also for introducing additional functionalities. In ferroelectric oxides, an accumulation of point defects at specific boundaries often deteriorates a polarization-switching capability, but on the one hand, delivers interface-driven phenomena. At present, it remains challenging to control oxygen vacancies at will to achieve a desirable defect structure. Here, we report a practical route to designing oxygen-vacancy distributions by exploiting the interaction with transition-metal dopants. Our thin-film experiments combined with *ab-initio* theoretical calculations for BiFeO_3_ demonstrate that isovalent dopants such as Mn^3+^ with a partly or fully electron-occupied *e*_g_ state can trap oxygen vacancies, leading to a robust polarization switching. Our approach to controlling oxygen vacancy distributions by harnessing the vacancy-trapping capability of isovalent transition-metal cations will realize the full potential of switchable polarization in ferroelectric perovskite oxides.

## Introduction

Spontaneous polarization (*P*_s_) in ferroelectrics presents unique opportunities to develop sensors, actuators, medical imaging transducers, and non-volatile random access memories^1–4^. Recently, ferroelectric tunnel junctions have attracted much attention owing to their potential applications in non-destructive readout memories in high-density integration^5–9^. In all these applications, the control of domain structures and polarization states by applying external fields are crucial to achieve desirable properties. Even though ferroelectric bulk has the insulating nature, charged domain walls stabilized by point defects act as an electrically conductive interface^10–15^, which opens possibilities for nanoelectronics such as domain-wall memories^16–18^. Moreover, exploiting a high mobility of defects enables to create a p–n junction that can be erased and inverted by electric fields^19^.

Meanwhile, an accumulation of defects at specific interfaces deteriorates the overall behaviour of polarization-switching dynamics^20,21^. For the charged domain walls stabilized by oxygen vacancies, the defect kinetics determine the switching process that is accompanied by a vacancy redistribution^22,23^, and thereby the device operation speed is limited by vacancy transport times^22^. The strong interaction with the vacancies gives rise to the clamping of domain walls^24–27^ and eventually causes imprint, retention loss, and fatigue of polarization states, preventing the widespread use of ferroelectric-based memories^1,21,28,29^.

For piezoelectric devices made of PbTiO_3_-based ferroelectrics, the properties can be tailored by doping of lower-valent and/or higher valent cations, mainly on the Ti^4+^ site, by adjusting the concentration of oxygen vacancies^30–33^. The doping of, e.g., Fe^3+^ increases an oxygen-vacancy concentration, and then the mobility of domain walls is reduced, leading to electromechanically hard lattices^32,34,35^. By contrast, the introduction of Nb^5+^ decreases the concentration and then the interaction between the domain walls and the vacancies is suppressed, resulting in soft lattices^32,36,37^. Despite over fifty years of intense research, it remains difficult to control the oxygen-vacancy distribution without changing its overall concentration. It is desirable to establish a design principle that can control and manipulate the vacant positions in the vicinity of the specific sites or interfaces at will with its controlled concentration.

## Interaction Between Transition-Metal Cations and Oxygen Vacancies

**Figure 1:**
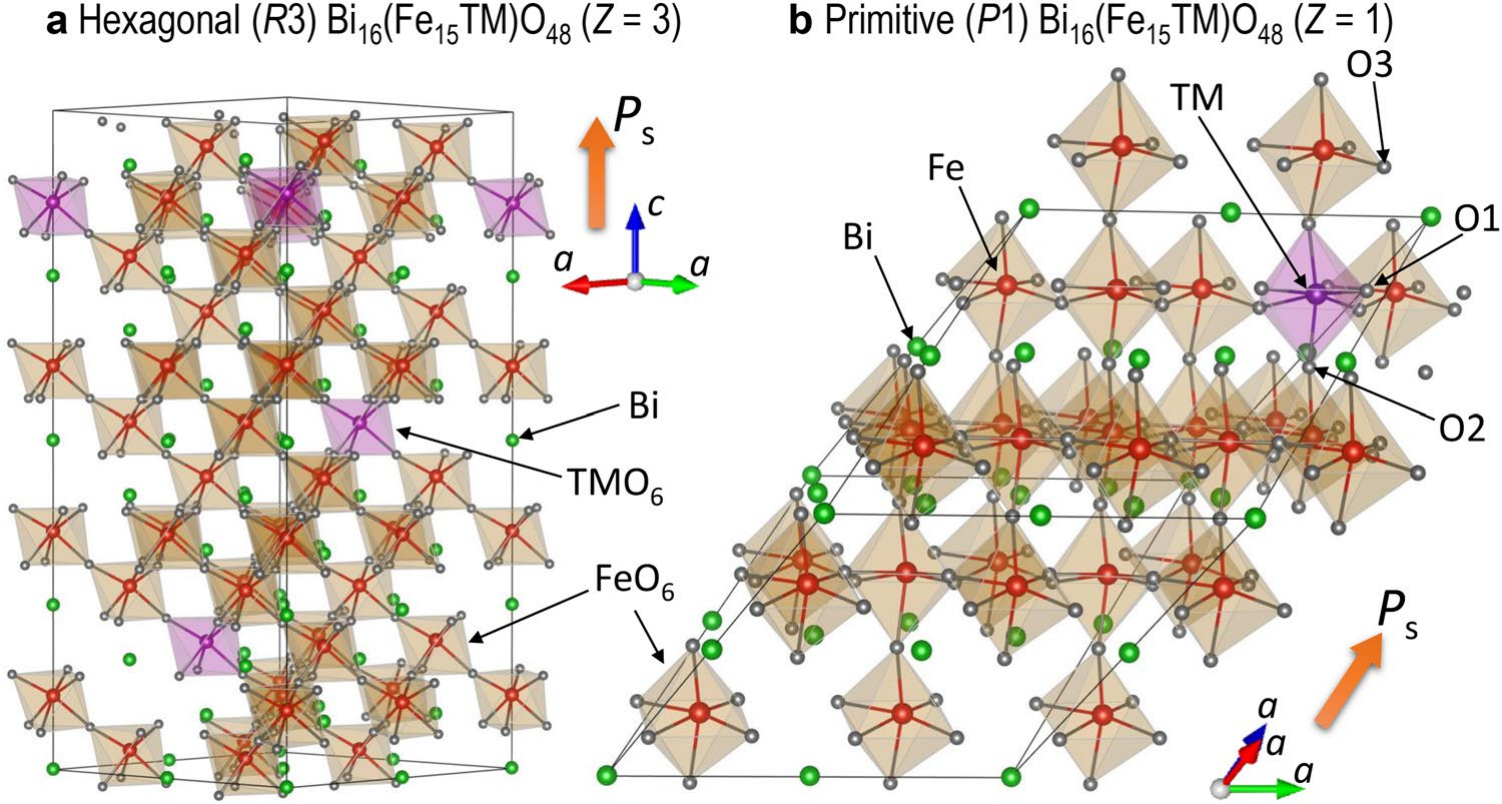
Crystal structures of transition-metal (TM)-doped BiFeO_3_. (**a**) Hexagonal Bi_16_(Fe_15_TM)O_48_ cell in space group *R*3 (*Z* = 3) and (**b**) primitive Bi_16_(Fe_15_TM)O_48_ in *P*1 symmetry (*Z* = 1) for DFT calculations. As TM atoms are positioned on the three-fold axis, TMs form the shortest bonds with three O1 atoms and the next-shortest bonds with three O2. O3 is the third nearest-neighbor oxygen atom with respect to TMs. For creating the defective cells with an oxygen vacancy, we remove one oxygen atom from the primitive cell and then perform the calculations for the Bi_16_(Fe_15_TM)O_47_ cell in *P*1 symmetry.

**Figure 2:**
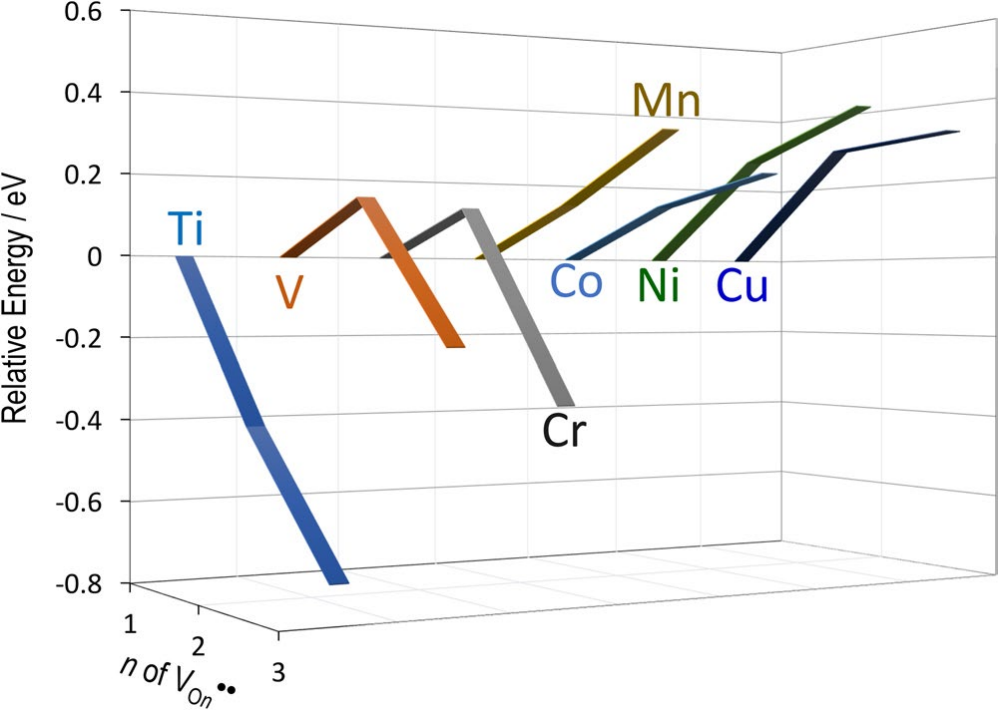
Total energies of transition-metal (TM)-doped BiFeO_3_. Total energy as a function of *n*, where *n* denotes the *n*-th nearest neighbor (NN) site of oxygen vacancy (*V*_O*n*_^••^) with respect to TM. As shown in Fig. 1, O1 and O2 are the 1^st^-NN and 2^nd^-NN oxygen atoms forming the TM-O polyhedron, respectively. The total energy of *n* = 1 is set at zero. A downward total energy with increasing *n* indicates that *V*_O_^••^ tends to keep away from TM atoms, whereas an upward total energy shows that *V*_O_^••^ is apt to be located close to the TM atoms.

**Figure 3:**
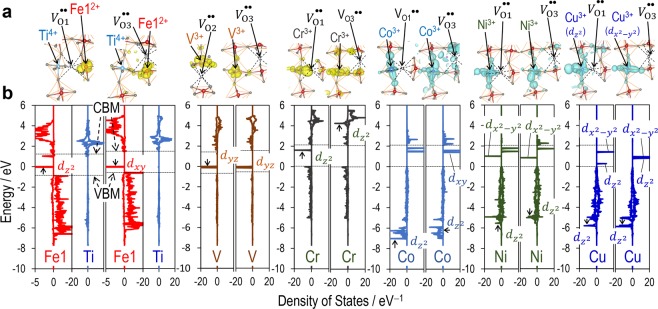
Electronic structures of transition-metal (TM)-doped BiFeO_3_. (**a**) Representative partial charge densities of the electronic states indicated by arrows in (**b**) total density of states (DOS) and partial DOS of TM atoms, where the states in the majority spin component (↑) are colored in blue while those in the minority spin component (↓) in yellow. In (**b**), the Fermi energy (*E*_F_) is set at zero, and upper and lower dashed lines are the conduction band minimum and the valence band maximum, respectively. For the TM = Ti cells, the DOS of the Fe1 adjacent to *V*_O*n*_^••^ is also shown. In each the DOS panel, the majority spin component (↑) is displayed in the right while the minority spin component (↓) in the left.

Figure 1 shows the crystal structures of transition-metal (TM)-doped cells for DFT calculations. For the primitive cell with an oxygen vacancy (*V*_O_^••^) in *P*1 symmetry (Fig. 1b), the total energy (*E*_total_) is calculated and compared; O1 and O2 are the oxygen atoms in the TM-O_6_ octahedron, and their distance with TMs is ~0.2 nm, while O3 is present in the next nearest FeO_6_ octahedra, and the distance of O3-TMs is lengthened to ~0.4 nm. Figure 2 displays the *E*_total_ of the defective cell with an oxygen vacancy (*V*_O*n*_^••^) on the *n*^th^ nearest-neighbor (NN) sites (*n* = 1−3) with respect to TMs, where the *E*_total_ of *n* = 1 is set at zero. Figure 3a,b depicts the total and (selected) partial density of states (DOS) with *V*_O1_^••^ (*V*_O2_^••^) and *V*_O3_^••^ along with the partial charge densities of the states indicated by arrows. The downward *E*_total_ with increasing *n* shows that the system is lower in energy when *V*_O_^••^ is away from TMs, while the upward *E*_total_ features an attractive interaction between TMs and *V*_O_^••^. In an ionic picture, TM^3+^ has the following electronic configuration: Ti^3+^ with *d*^1^, V^3+^ with *d*^2^, Cr^3+^ with *d*^3^, Mn^3+^ with *d*^4^, Co^3+^ with *d*^6^, Ni^3+^ with *d*^7^, Cu^3+^ with *d*^8^. The valence states of TMs expect for Ti, Co, and Cu are confirmed by the partial magnetic moment analysis^38^: V^3+^ with ~1.8 *μ*_B_, Cr^3+^ with ~2.8 *μ*_B_, Mn^3+^ with ~3.6 *μ*_B_, Ni^3+^ with ~0.8 *μ*_B_, where *μ*_B_ is the Bohr magneton. We note that the cells of TM = Ti, V, and Cr have a smaller *E*_total_ of *n* = 3 than that of *n* = 1, whereas those of others exhibit the smallest *E*_total_ with *n* = 1. These tendencies reflect the following spin configuration of TM^3+^: the *e*_g_ state for the cells of Ti, V, and Cr is empty, while that for others is partly or fully electron-occupied. The detailes of the formation energies of TM dopants in the trivalent ionic state into BiFeO_3_ have been reported by Gebhardt and Rappe^39^.

The results described above can be intuitively explained by the electronic states formed by the orbital interaction between TM-3*d* and adjacent O-2*p*. For TM = Ti, the system has Ti^4+^ (*d*^0^) and Fe1^2+^ (*d*^6^) because of the higher energy states of Ti-3*d*, where the Fermi level (*E*_F_) is located at the electron-occupied Fe1-3*d* state. These results are supported by the partial magnetic moments of 0.1 *μ*_B_ for Ti-3*d* and 3.6 *μ*_B_ for Fe1-3*d* (smaller than the other Fe-3*d* values of ~4.1 *μ*_B_). This can be qualitatively understood by the charge transfer: the electron of Ti^3+^ is transferred to Fe1 on the 1^st^ NN site with the oxygen vacancies. With respect to the valence band maximum (VBM; the lower dashed line), the Fe1-*d*_*xy*_ state with *V*_O3_^••^ is lower by ~0.1 eV than the Fe1-*d*_*z*2_ state with *V*_O1_^••^, which is the main reason why the *n* = 3 cell is lower in energy than the *n* = 1 cell. The V cells have two occupied states of V-3*d* (*d*^2^) in the gap; comparing the higher gap states at the *E*_F_, the *d*_*yz*_ state with *V*_O3_^••^ is lower in energy by ~0.1 eV than the *d*_*yz*_ state with *V*_O2_^••^, resulting in the lower *E*_total_ of *n* = 3. For TM = Cr, the empty gap state with the *d*_*z*2_ character appears with *V*_O1_^••^, whereas there is no state in the gap with *V*_O3_^••^. Although the change in the coordinate from CrO_6_ (*n* = 3) to CrO_5_ (*n* = 1) splits the *e*_g_ state into the low-lying *d*_*z*2_ and the high-lying *d*_*x*2−*y*2_, this does not lead to a significant stabilization in the system, because both the states are empty. The CrO_6_ structure (*n* = 3) maintains a regular octahedron, and the Cr-3*d* state is represented by *t*_2g_^3^*e*_g_^0^, which contributes to a lower *E*_total_ of *n* = 3. A stabilization of *V*^••^ away from Cr has also been reported for hexagonal BaTiO_3_^40^. The details for TM = Mn are described later.

For TM = Co, the electronic configuration of Co^3+^ (*d*^6^) in *O*_*h*_ symmetry is expressed as *t*_2g_^4^*e*_g_^2^ in the high-spin state^41,42^. Regardless of the position of *V*^••^ ^40^, the occupied DOS of Co-3*d* in the majority spin component (↑) is spread in the valence band (−5 to 0 eV), showing that one electron is present in the bonding state of *e*_g_(↑). In this case, the electronic state is regarded as *t*_2g_(↓)^3^*e*_g_(↓)^2^*e*_g_(↑)^1^ rather than as *t*_2g_(↓)^3^*e*_g_(↓)^2^*t*_2g_(↑)^1^, in which the strong hybridization of Co-*e*_g_ with O-2*p* leads to a relatively small magnetic moment of Co^3+^ with ~3.0 *μ*_B_. Comparing the energies of the *d*_*z*2_-derived state (↓), we found that the cell with *V*_O1_^••^ has the low-lying state at the valence band minimum of −7.1 eV, which is mainly due to the absence of O1 (the presence of *V*_O1_^••^), along with the orbital mixing with O-2*p* of two apical oxygen atoms. This marked stabilization of the *d*_*z*2_-derived bonding state is attributed to the lower *E*_total_ of *n* = 1. The Ni cells exhibit relatively complex electronic features; the electronic configuration of Ni^3+^ (*d*^7^) in *O*_*h*_ symmetry is described as *t*_2g_^6^*e*_g_^1^. With *V*_O1_^••^ in the minority spin component (↓), the unoccupied *d*_*x*2−*y*2_ state is present in the gap, while the *d*_*z*2_-derived state is markedly stabilized and appears at the valence band minimum because of the strong hybridization with the adjacent O-2*p*. Although the similar feature was found in that with *V*_O3_^••^, its energy is higher by ~0.5 eV. The low-lying *d*_*z*2_-derived state with *V*_O1_^••^ is responsible for the lower *E*_total_ of *n* = 1. For TM = Cu, the electronic configuration of Cu^3+^ (*d*^8^) is expressed as *t*_2g_^6^*e*_g_^2^, which is supported by the empty *e*_g_(↑) state present in the band gap. Nevertheless, the apparent orbital mixing of Cu-*e*_g_(↑) and O-2*p* is clearly seen in their partial charge density (Fig. 3a) and thus result in a relatively small magnetic moment of Cu^3+^ with 0.6–0.8 *μ*_B_. The cell with *V*_O3_^••^ has the *e*_g_(↑) state whereas that with *V*_O1_^••^ features the low-lying *d*_*z*2_ and the high-lying *d*_*x*2−*y*2_ sates owing to the absence of O1. Also for the occupied states in the minority spin component (↓), the cell with *V*_O1_^••^ has the *d*_*z*2_-derived state at ~−6.1 eV, which is lower by ~0.3 eV than that with *V*_O3_^••^, leading to the lower *E*_total_ of *n* = 1.

### Oxygen-vacancy distributions

**Figure 4:**
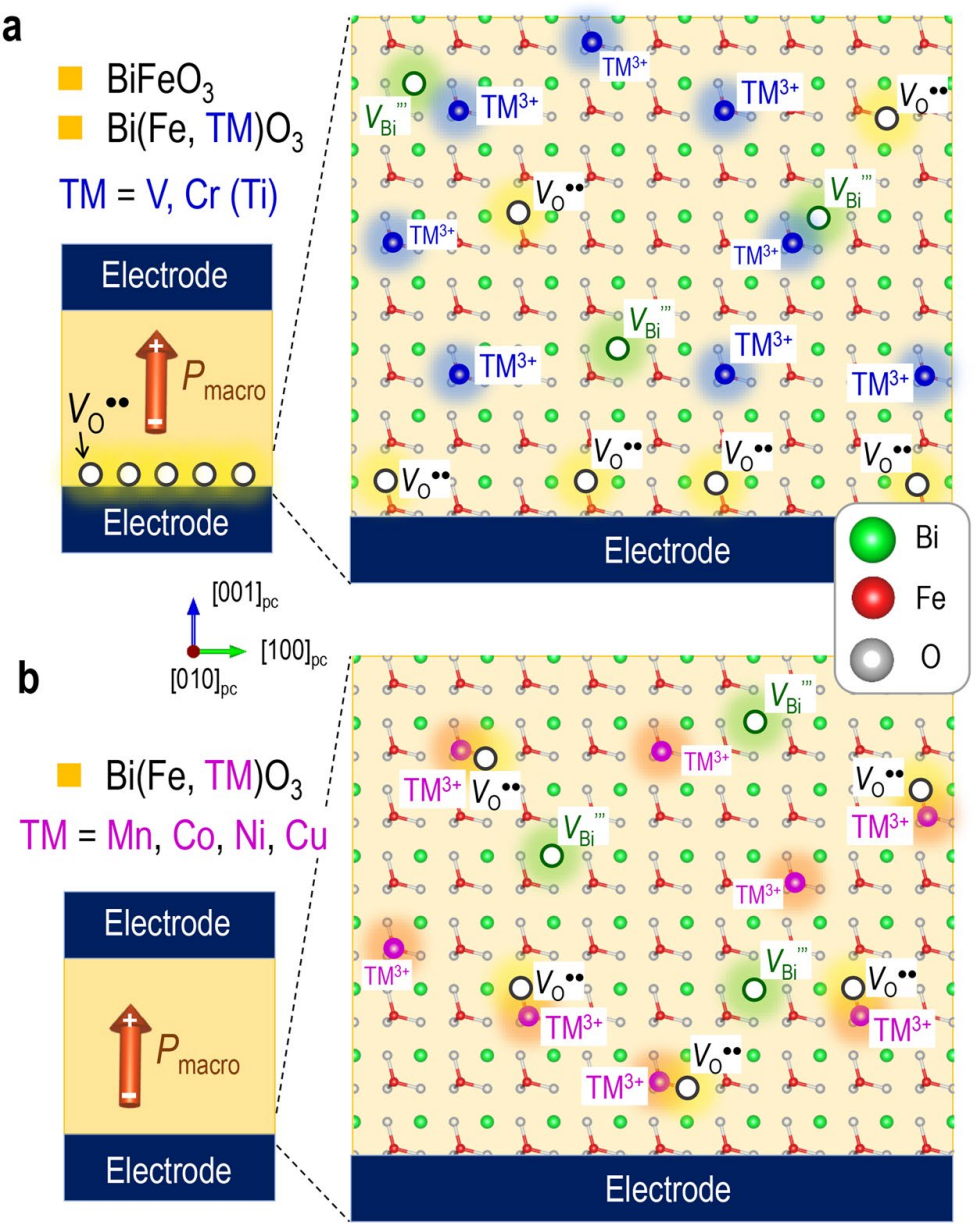
Oxygen-vacancy distributions in capacitor form. (**a**) BiFeO_3_ and transition-metal (TM)-doped BiFeO_3_ [Bi(Fe,TM)O_3_, TM = V, Cr (Ti)] and (**b**) TM-doped BiFeO_3_ [Bi(Fe,TM)O_3_, TM = Mn, Co, Ni, Cu]. The TM atoms except for Ti have a valence state of TM^3+^. As the TM = Ti cell exhibits a valence state of Ti^4+^ because of the presence of Fe^2+^, Ti is indicated in parenthesis in (**a**). In (**a**), oxygen vacancy (*V*_O_^••^) does not find a stable site inside the lattice and thereby accumulates at the interface with the bottom electrode, forming a *V*_O_^••^-rich layer; this defective layer is formed by an attractive interaction with negative bound charges caused by a discontinuity of spontaneous polarization (*P*_s_). In (**b**), *V*_O_^••^ is stabilized adjacent to TM^3+^; provided that the concentration of TM is higher than that of *V*_O_^••^ ([*V*_O_^••^]), all of *V*_O_^••^ are trapped by TM^3+^.

Figure 4 shows the *V*_O_^••^ distributions of the TM-doped cells. For TM = Ti, V, and Cr, *V*_O_^••^ does not find an energetically favourable site inside the lattice; the vacancies in the vicinity of the bottom electrode are pulled toward its interface owing to a strong depolarization field arising from the discontinuity of *P*_s_, suggesting a formation of an *V*_O_^••^-rich layer (Fig. 4a). This defective layer has been reported for non-doped BiFeO_3_ films^22,43–46^. By contrast, the cells with TM = Mn, Co, Ni, and Cu provide a stable position of *V*_O_^••^, i.e., the 1^st^ NN site adjacent to the TMs. Provided that the concentration of TMs is higher than that of *V*_O_^••^ and also that the attractive interaction between *V*_O_^••^ and TMs is sufficiently strong, *V*_O_^••^ is trapped by TMs in an equilibrium state, as displayed in Fig. 4b.

For a robust switching of *P*_s_ by applying electric fields, in addition to the attractive interaction of *V*_O_^••^-TM, the doped lattice should meet the following requirements: the first is a high solubility limit of TM; the second is the stable valence state of TM^3+^; and the third is the electronic character of TM that does not lead to a significant increase in leakage current. Here, we choose Mn as a TM because of the following reasons: Mn can be introduced into the lattice in a wide composition range^47^, and the valence state is controllable to Mn^3+^ ^48^. Moreover, the empty *d*_*x*2−*y*2_ state of the Mn cell with *V*_O1_^••^ is far above the VBM^49^, and thereby a leakage current is expected to be relatively low.

### Crystal structures and properties

**Figure 5:**
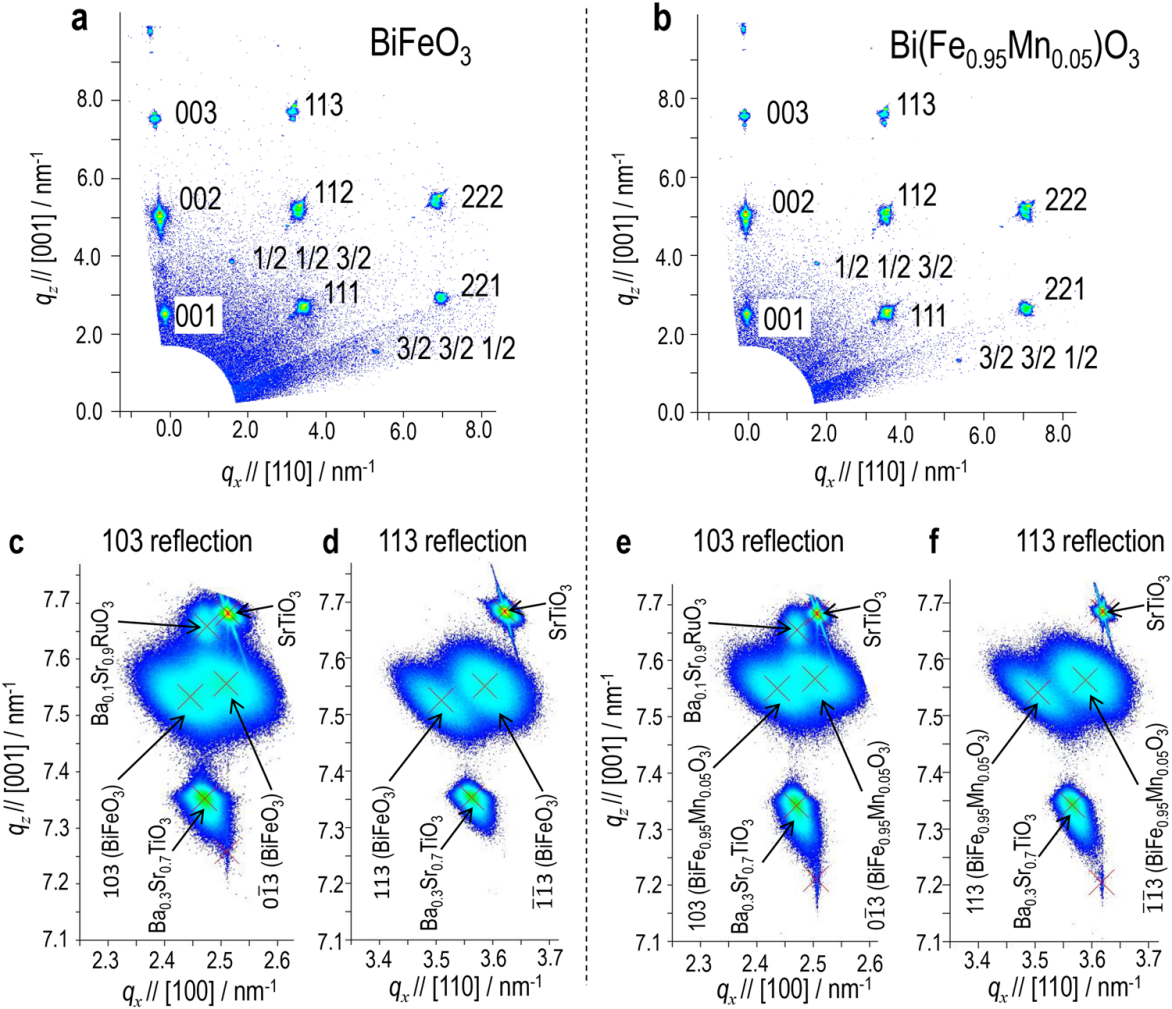
X-ray diffraction analysis. X-ray diffraction reciprocal space maps for (**a**,**c**,**d**) BiFeO_3_ and (**b**,**e**,**f**) Bi(Fe_0.95_Mn_0.05_)O_3_ capacitors with a Ba_0.3_Sr_0.7_TiO_3_ (300 nm)-buffered Ba_0.1_Sr_0.9_RuO_3_ electrodes. (**a**,**b**) Maps measured using the sources of Cu-*K*α + *K*β while (**c**,**d**,**e**,**f**) those measured using Cu-*K*α_1_. All the Mirror indices and crystallographic directions are described in the pseudo-cubic notation; ‘pc’ is omitted.

As displayed in Fig. 5 of the X-ray diffraction reciprocal space maps (XRD-RSMs), we confirmed that the capacitors are hetero-epitaxially grown on the SrTiO_3_ substrate, *i*.*e*., the [001]_pc_ directions of the films are normal to (001) of the substrate. For both the capacitors, we observed the superlattice reflections of 1/2 1/2 3/2 and 3/2 3/2 1/2 in addition to the fundamental ones. Figure 5c–f displays the high-resolution XRD-RSMs of the 103 _pc_ and 113 _pc_ reflections. The Ba_0.3_Sr_0.7_TiO_3_ film exhibits the reflection with a smaller *q*_*x*_ value compared with the substrate. We note that the *q*_*x*_ position of the reflection for the Ba_0.1_Sr_0.9_RuO_3_ electrode is almost the same as that of the Ba_0.3_Sr_0.7_TiO_3_ film. These results show that the Ba_0.1_Sr_0.9_RuO_3_ electrode along with the Ba_0.3_Sr_0.7_TiO_3_ film acts as a buffer layer for reducing the lattice mismatch between BiFeO_3_ (BFO) or Bi(Fe_0.95_Mn_0.05_)O_3_ (Mn-BFO) and SrTiO_3_.

The 103 and 113 reflections are split into two spots, which is the typical character of the rhombohedral structure in *R*3*c* space group. From the peak positions of these spots, we obtained the following rhombohedral lattice parameters for the BFO film: *a* = 0.3984 nm, *α* = 89.53 deg., and *V* (lattice volume) = 6.32 × 10^−2^ nm^3^, which are almost the same as those of the bulk (*a* = 0.3964 nm, *α* = 89. 43 deg. and *V* = 6.23 × 10^−2^ nm^3^)^50^. Because a monoclinic distortion induced by compressive stress was not found from the XRD data and the difference in the parameter *a* is as small as 0.5%, we think that a strain-free bulk-like lattice is obtained for the BFO film. The Mn-BFO film has *a* = 0.3978 nm, *α* = 89.59 deg., and *V* = 6.29 × 10^−2^ nm^3^, indicating that the 5% Mn doping does not lead to a significant change in crystal structure and that a bulk-like lattice is also attained for the Mn-BFO film.

**Figure 6:**
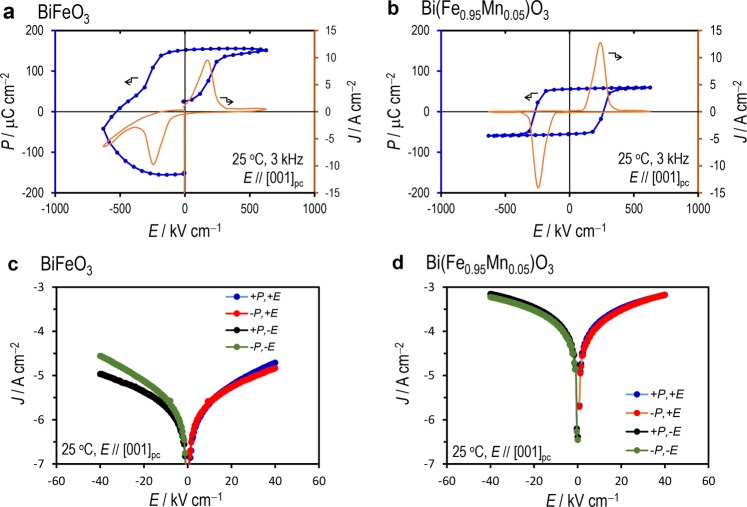
Polarization and leakage-current properties. (**a**,**b**) Polarization-electric field (*P-E*) hysteresis loops (3 kHz) and (**c**,**d**) leakage-current density as a function of *E* (*J*-*E*) properties along the [001]_pc_ direction; (**a**,**c**) BiFeO_3_ and (**b**,**d**) Bi(Fe_0.95_Mn_0.05_)O_3_ capacitors with a Ba_0.3_Sr_0.7_TiO_3_ (300 nm)-buffered Ba_0.1_Sr_0.9_RuO_3_ electrodes. Data were measured at 25 °C.

Figure 6a,b shows the polarization-electric field (*P-E*) hysteresis properties with the current profiles during the polarization switching. For the BFO capacitor, the *P* curve in a positive field exhibits a loop typical for normal ferroelectrics, whereas that in a negative field rounds. This feature is attributed to a markedly large leakage current at negative fields. The asymmetric hysteresis loop has been reported for as-prepared BFO films in a strained state^44,51,52^. We note that the Mn-BFO capacitor presents a well-saturated hysteresis loop with a remanent polarization of 55.4 *μ*C cm^−2^, where the *P-E* loop, as well as the current profile, is symmetric with respect to *E*. This *P*_r_ value along [001]_pc_ provides a *P*_s_ (// [111]_pc_) of 96.0 μC cm^−2^, which is close to the *P*_s_ observed for bulk crystals^53^ and calculated by first-principles calculations^54^. Figure 6c,d displays the current density-electric field (*J-E*) properties in the low field region (−50 kV cm^−1^ to 50 kV cm^−1^). The BFO capacitor exhibits a rectification behavior, where the *J* becomes larger when the *E* direction is parallel to the macroscopic polarization. In particular, the negative *P* state (−*P*) at negative fields (−*E*) features a markedly large *J*, which is consistent with the *P-E* properties. Even though the Mn-BFO capacitor has a higher *J* by over an order of magnitude, it presents a symmetric *J*-*E* property. The rectified current behavior and the associated round *P-E* loop observed for the BFO capacitor can be explained by an *V*_O_^••^-rich layer at the BFO/electrode interface (Fig. 4a)^22,43–46^, which is probably formed during either the film deposition or the cooling process^55^.

### Electronic structures of Mn-doped BiFeO_3_

**Figure 7:**
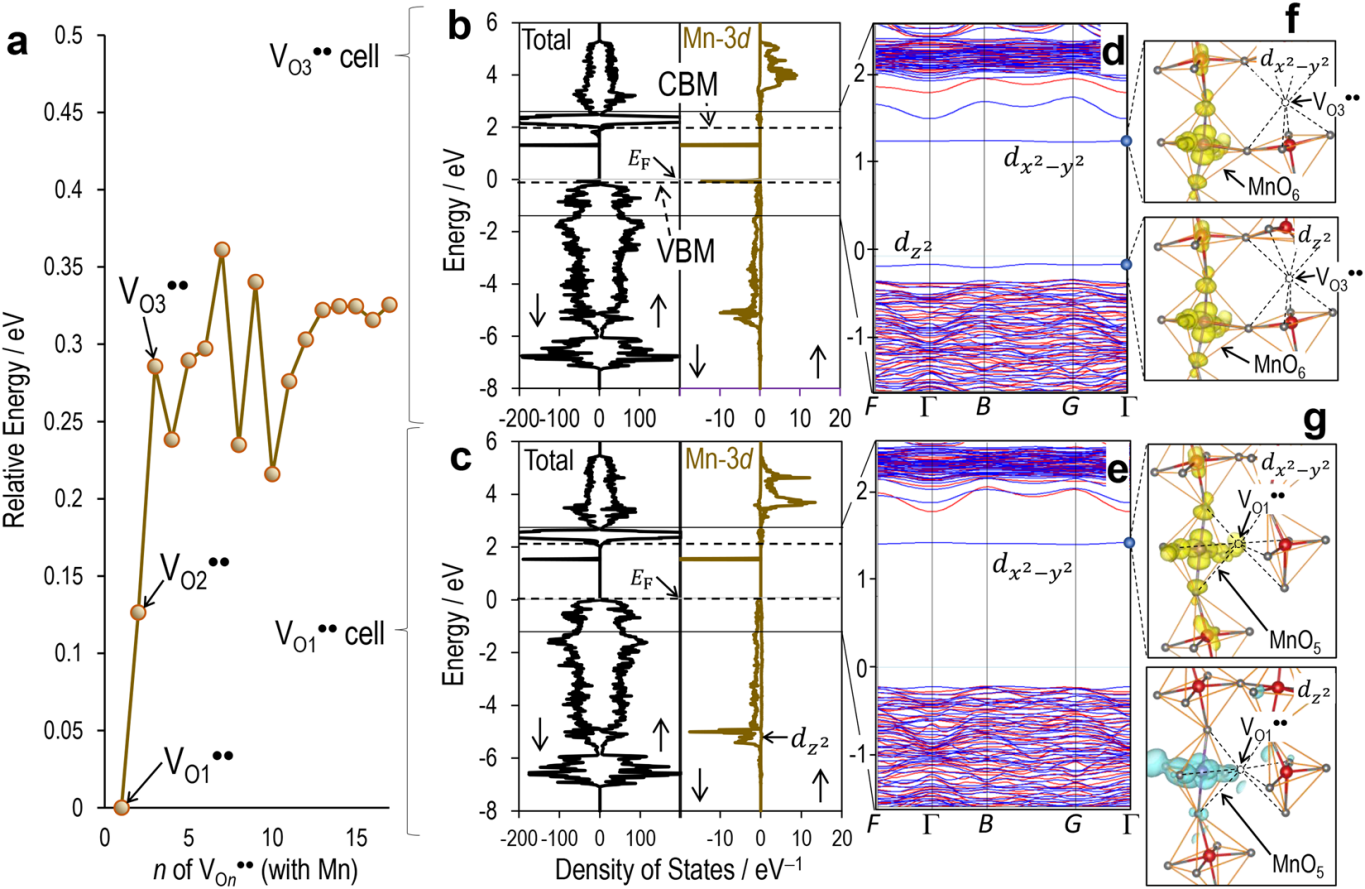
Interaction between Mn and Oxygen vacancy. (**a**) Total energy as a function of *n* obtained by DFT calculations, where *n* denotes the *n*-th nearest neighbor (NN) site of oxygen vacancy (*V*_O*n*_^••^) with respect to Mn^3+^. O1 and O2 are the 1^st^-NN and 2^nd^-NN oxygen atoms (see in Fig. 1b). The total energy of *n* = 1 is set at zero. Total density of states (DOS) and partial DOS of the cells of (**b**) *V*_O3_^••^ and (**c**) *V*_O1_^••^ and their respective band structures are shown in (**d**,**e**). In DOS panels, the majority spin component (↑) is indicated in the right while the minority spin component (↓) in the left. The Fermi energy (*E*_F_) is set at zero, and upper and lower dashed lines are the conduction band minimum and the valence band maximum, respectively. In band structures, the majority spin component is colored in red and the minority spin component in blue. Partial charge densities of the gap states formed primarily by the orbital interaction between Mn-3*d* and O-2*p* for the (**f**) *V*_O3_^••^ and (**g**) *V*_O1_^••^ cells, where the majority spin components are colored in blue while those in the minority spin component in yellow. The lower panel of (**g**) is the Mn-3*d* derived state in the valence band indicated by an arrow in (**c**).

Figure 7 presents the results of the DFT calculations for the defective cells of TM = Mn. The relative value of *E*_total_ as a function of *n* is plotted in Fig. 7a. We note that the cells of *n* greater than three exhibit a relatively large *E*_total_ by 0.1–0.2 eV compared with *n* = 1 and 2, indicating that the cell has a lower energy provided that *V*_O_^••^ is located adjacent to Mn. Notably, the energy gain when *V*_O_^••^ is present on the O1 (*n* = 1) site is 250 meV, which is three times as large as that of the thermal energy *k*_B_*T* even at the deposition temperature (*T*_sub_ = 610 °C, *k*_B_*T*_sub_ ~75 meV). Because the Curie temperature (830 °C for BiFeO_3_) is higher than *T*_sub_, a crystallization during the film deposition occurs in the ferroelectric state. We assume that the majority of *V*_O_^••^ are trapped by Mn even in the deposition process at high temperatures through a trapping-detrapping dynamics^56,57^. Also, after the film is exposed to a subsequent annealing or a polarization switching, Mn provides the preferred site for *V*_O_^••^ in its immediate vicinity, and then almost all *V*_O_^••^ associates with Mn in an equilibrium state.

Here, we address the mechanism of the attractive interaction between Mn and *V*_O_^••^ (Fig. 4b). Given that Mn is octahedrally coordinated with six oxygen atoms and is placed in *O*_*h*_ symmetry, Mn^3+^ (*d*^4^) has a configuration of *t*_2g_^3^*e*_g_^1^, and an electron occupies the half of the *e*_g_ state. In the ferroelectric state with *V*_O_^••^, the degeneracy of *e*_g_ is lifted, rendering either of the component *d*_*x*2−*y*2_ or *d*_*z*2_ filled with electron. Therefore, the total energy of the system is governed by an energy lowering of the highest-occupied *d* level as a result of the interaction with neighbors.

Figure 7b,c displays the density of states (DOS) of the *V*_O3_^••^ and *V*_O1_^••^ cells, and their respective band structures are presented in d and e. The fundamentals of the electronic structures are described in the literature^44,49,54^. For the *V*_O1_^••^ cell having the smallest *E*_total_, an empty state derived mainly from the Mn-3*d* state (*d*_*x*2−*y*2_) is left in the band gap, i.e., an unoccupied gap state is formed in the minority spin component above the *E*_F_. The relatively large DOS of Mn-3*d* around −5 eV indicates that the rest four occupied *d* states (*d*_*xy*_, *d*_*xz*_, *d*_*yz*_, and *d*_*z*2_) exist inside the valence band, which are hybridized with the neighboring O-2*p* (e.g., the *d*_*z*2_-derived partial charge density depicted in the lower panel of Fig. 7g). By contrast, the *V*_O3_^••^ cell has two gap states: one is an empty *d*_*x*2−*y*2_ state and the other is an occupied *d*_*z*2_ state. This result proves that the *d*_*z*2_-derived occupied state is much lower in the *V*_O1_^••^ cell than in the *V*_O3_^••^ one.

### Oxygen-vacancy trapping

When *V*_O_^••^ associates with Mn in the immediate vicinity, a MnO_5_ pyramid is formed, and Mn-3*d* interacts strongly with O-2*p* of the adjacent five oxygen atoms. Because a repulsive Coulomb interaction between electrons is markedly reduced along the Mn-*V*_O_^••^ direction, the MnO_5_ pyramid can accommodate the *d*_*z*2_ orbital affordably (see the partial charge density in Fig. 7g), and thereby its level is lower in energy far below *E*_F_. Provided that *V*_O_^••^ is away from Mn, which involves a change from the MnO_5_ pyramid to the MnO_6_ octahedron, the *d* orbitals are affected by the adjacent six oxygens and then the *d*_*z*2_ orbital is shifted to higher energy, forming the occupied gap state above the VBM. As a result, the system is stabilized when *V*_O_^••^ resides adjacent to Mn. These results lead to the conclusion that Mn acts as an effective trap for *V*_O_^••^.

We address the *V*_O_^••^ distribution in the capacitors. As reported in the literature^58^ and shown in Fig. 6, the as-prepared BFO capacitor show the distinct characteristics such as the asymmetric *P*-*E* hysteresis loop and the rectified current behavior. All these properties can be explained by an *V*_O_^••^-rich layer formed at the BFO/electrode interface (Fig. 3a). During the deposition process at high temperatures, BFO tends to have a Bi-poor composition because of a high vapor pressure of Bi^46,59,60^; Bi vacancy (*V*_Bi_″′) is formed and acts as an acceptor^13,44^, which is accompanied by *V*_O_^••^ for charge compensation^61,62^. Atomic-scale chemical and structural analyses show that Fe^4+^ (Fe_Fe_^•^) is abundant in the domain wall region in BiFeO_3_^13^. DFT calculations reveal that the iron atom adjacent to *V*_O_^••^ tends to a valence state of Fe^4+^ stabilized by a FeO_5_ pyramid^13^, which can explain a high concentration of Fe^4+^ owing to an accumulation of *V*_O_^••^ at the domain walls. We therefore think the charge neutrality of [*V*_Bi_″′] ~ [*V*_O_^••^] ~ [Fe_Fe_^•^] and express the overall defect formation by

1 $${{{\rm{Bi}}}_{{\rm{Bi}}}}^{\times }+{{{\rm{Fe}}}_{{\rm{Fe}}}}^{\times }+{{{\rm{O}}}_{{\rm{o}}}}^{\times }\to {V\prime\prime\prime }_{{\rm{Bi}}}+{{{\rm{Fe}}}_{{\rm{Fe}}}}^{\cdot }+{{V}_{{\rm{o}}}}^{\cdot \cdot }+{\rm{Bi}}({\rm{g}})+1/2({\rm{g}}),$$

where Bi_Bi_^×^, Fe_Fe_^×^, and O_O_^×^ are Bi^3+^ on the Bi site, Fe^3+^ on the Fe site, and O^2−^ on the O site, respectively. Provided that *V*_O_^••^ hops to the other O site, the 1^st^ NN Fe atom adjacent to the newly created *V*_O_^••^ is oxidized from Fe^3+^ to Fe^4+^ as a result of an electron transfer. Therefore, Fe_Fe_^•^ always associates with *V*_O_^••^ regardless of its position and is present as the defect complex of *V*_O_^••^-Fe_Fe_^•^, which leads us to propose that the charge neutrality is expressed by [*V*_Bi_″′] ~ [*V*_O_^••^-Fe_Fe_^•^]^44^. Because the mobility of *V*_O_^••^ is several orders of magnitude higher than that of cation defects such as *V*_Bi_″′^25^, we can consider that *V*_Bi_″′ is frozen, except at high temperatures during the film deposition, and thus has a random distribution at low temperatures. By contrast, *V*_O_^••^ is mobile even at room temperatures^19^ and then accumulates if its preferred site exists. Although the negatively charged *V*_Bi_″′ is supposed to attract *V*_O_^••^ owing to an electrostatic interaction, DFT calculations^44^ show that *V*_Bi_″′ does not act as a trap of *V*_O_^••^ and predict that *V*_O_^••^ is randomly distributed in bulk form. It has been reported that *V*_O_^••^ is apt to accumulate at high-energy boundaries such as ferroelastic domain (twin) walls and ferroelectric/electrode interfaces^10–15,30–33^.

As described above, we think that the ferroelectric films are crystallized in the polar state in their deposition process; the films have ferroelectric polarization once the BFO lattice is constructed. In capacitor form, the discontinuity of the *P*_s_ vector is inevitable at interfaces with electrodes and results in a depolarization field^22,43–46^. As the *P*_s_ of BFO is markedly large, the depolarization field becomes strong. We note that the tail of *P*_s_ vector has negative bound charges that attract positively charged *V*_O_^••^. DFT calculations^51^ predict that the *V*_O_^••^ near the electrodes moves to the interface and forms an *V*_O_^••^-rich layer to reduce an electrostatic energy. The concentration of *V*_Bi_″′ ([*V*_Bi_″′]) is likely to be less than 3%^63^, and leads to a comparable [*V*_O_^••^] at most because of [*V*_Bi_″′] ~ [*V*_O_^••^-Fe_Fe_^•^]. In the Mn-BFO capacitor, *V*_O_^••^ can associate exclusively with Mn because of its higher content (5% Mn), as depicted in Fig. 3b. We conclude that Mn acts as an effective trap of *V*_O_^••^ and thereby inhibits the formation of an *V*_O_^••^-rich layer at the interface.

## Discussion

We show that isovalent dopants with partly or fully electron-filled *e*_g_ state, such as Mn^3+^ (Mn_Fe_^×^), act as an effective trap for oxygen vacancies, which enables us to provide a design principle to tailor the defect structures in a wide range of [*V*_O_^••^]. The isovalent dopants do not influence [*V*_O_^••^], and the intrinsic defect of *V*_Bi_″′ dictates [*V*_O_^••^], where [*V*_Bi_″′] is not easily controllable. Introducing higher valent cations such as Ti^4+^ (Ti_Fe_^•^) reduce [*V*_O_^••^]^64^; the doping of a small amount of Mn_Fe_^×^ together with [Ti_Fe_^•^] ( > 3[*V*_Bi_″′]) can lower [*V*_O_^••^] by several orders of magnitude, and all the vacancies are present only in the adjacent vicinity of Mn^3+^. This defect structure leads to a high mobility of domain walls, which is suitable not only for reliable high-speed non-volatile memories but also for piezoelectric applications utilizing high strain constants. For the applications based on conducting domain walls stabilized by *V*_O_^••^, a finely tuned [*V*_O_^••^] should considerably improve the device performance, which is accomplished by the balanced co-doping of Mn_Fe_^×^ and Ti_Fe_^•^; free, mobile *V*_O_^••^ with an adjusted concentration accumulates the domain walls and leads to a desirable interaction strength, delivering domain-wall memories exhibiting a high-speed switching.

We expect that the application of the design principle of defect structures to other (multi)ferroic materials can provide a practical route to controlling and manipulating oxygen-vacancy distributions by harnessing the vacancy-trapping capability of isovalent transition-metal cations in ferroelectric perovskite oxides.

## Methods

### Experiments

Thin films of ferroelectric BiFeO_3_ (BFO) and Mn(5%)-substituted BiFeO_3_ [Mn-BFO, Bi(Fe_0.95_Mn_0.05_)O_3_] were fabricated on (100) SrTiO_3_ single-crystal substrates. To reduce a lattice mismatch between the substrate and the ferroelectric films as much as possible, we adopted Ba_0.3_Sr_0.7_TiO_3_ as a buffer layer and Ba_0.1_Sr_0.9_RuO_3_ as an electrode. We prepared the capacitors of Ba_0.1_Sr_0.9_RuO_3_ (30 nm)/BiFeO_3_ or Bi(Fe_0.95_Mn_0.05_)O_3_ (125 nm)/Ba_0.1_Sr_0.9_RuO_3_ (30 nm)/ Ba_0.3_Sr_0.7_TiO_3_ (300 nm)/SrTiO_3_, where their thickness is indicated in parenthesis. All the films were deposited hetero-epitaxially by pulsed-laser deposition (PLD). The Ba_0.3_Sr_0.7_TiO_3_ buffer layer was grown at a substrate temperature (*T*_sub_) of 740 °C under 0.26 Pa O_2_ atmosphere with a laser repetition rate of 1 Hz. The Ba_0.1_Sr_0.9_RuO_3_ electrodes were prepared at a *T*_sub_ of 610 °C under 13 Pa O_2_ atmosphere with a laser repetition rate of 1 Hz. For the ferroelectric layers, *T*_sub_, oxygen pressure and a laser repetition rate were set at 640 °C, 2.6 Pa, and 7 Hz, respectively. Then, the Ba_0.1_Sr_0.9_RuO_3_ top electrode was prepared in the same manner as the bottom one. After the deposition process, the capacitors were annealed in air at 450 °C for one hour.

We characterized the capacitors in the as-prepared state and did not employ any treatment such as an electrical training^43,44^ to control the distribution of oxygen vacancy (*V*_O_^••^). Crystal structure analysis was performed by X-ray diffraction (XRD) reciprocal space mapping (RSM), where the sources of Cu-*K*α + *K*β and Cu-*K*α_1_ were used for wide-area and small-area (high-resolution) RSMs, respectively. The polarization-electric field (*P-E*) properties were measured at 25 °C, where the upward (downward) electric field and polarization are expressed as + *E* (−*E*) and +*P* (−*P*). For example, the vector of +*E* or +*P* is directed from the bottom to the top electrodes. We adopted the pseudo-cubic notation (denoted by ‘pc’) throughout this paper.

### DFT Calculations

First-principles calculations based on DFT^65^ were performed within the generalized gradient approximation (GGA)^66^ in the projector-augmented-wave (PAW) method^67^, as implemented in the Vienna *ab initio* simulation package (VASP)^68^. We employed the gradient corrected exchange-correlation functional of the Perdew-Burke-Ernzerhof revised for solids (PBEsol)^69^. Within the simplified GGA + *U* approach^70^, we added on-site Coulomb interaction parameters of *U*−*J* = 4 eV to all the *d* orbitals of transition metal (TM) atoms. Because the magnetic structure of BFO can be approximated to a *G*-type antiferromagnetism, we considered an antiferromagnetic spin configuration formed by the *d* electrons. Before geometry optimizations of BFO, we put a magnetic moment of +5 *μ*_B_ (*μ*_B_ denotes the Bohr magneton) or −5 *μ*_B_ to the Fe atoms, which is accompanied by a symmetry lowering: the space group changes from *R*3*c* to *R*3.

For TM-substituted cells (TM = Ti, V, Cr, Mn, Co, Ni, Cu), we transformed the optimized BiFeO_3_ cell with *R*3 symmetry (*Z* = 6) to the primitive rhombohedral lattice (Bi_2_Fe_2_O_6_, *Z* = 1) and then created the supercell of 2 × 2 × 2, leading to the Bi_16_Fe_16_O_48_ structure. We replaced one Fe atom with a negative magnetic moment by TM [Bi_16_(Fe_15_TM)O_48_] and lifted the symmetry to the hexagonal *R*3 [TM-BiFeO_3_ cell (*Z* = 3), see Fig. 1a]. This supercell was geometrically optimized with a Monkhorst-Pack Γ-centred *k*-point mesh of 3 × 3 × 3. All results were obtained by treating the following valence electrons: 5*d*, 6*s*, and 6*p* for Bi, 3*p*, 3*d*, and 4*s* for Ti, V, Cr, and Mn, 3*d* and 4*s* for Fe, Co, Ni, and Cu, and 2*s* and 2*p* for O. The plane-wave cut-off energy was set at 520 eV in all calculations.

In the TM-BiFeO_3_ cells, TM is positioned on the three-fold axis in *R*3 symmetry, and seventeen kinds of oxygen atom having different bond lengths with TM exist. After we created the primitive lattice of the TM-BiFeO_3_ cell [Bi_16_(Fe_15_TM)O_48_ (*Z* = 1), Fig. 1b], we constructed 17 defective TM-BiFeO_3_ cells with one *V*_O_^••^, Bi_16_(Fe_15_TM)O_47_, in *P*1 symmetry and then relaxed the fractional coordinates of all the atoms in the fixed cell size, where the Γ-centred 3 × 3 × 3 *k*-point mesh was also used. For these calculations, the valence state of TM remained to be +3 by controlling the total number of electrons. It has been reported that oxygen vacancies in zinc ferrites change the iron-iron coupling from the antiferromagnetic to the ferromagnetic spin configuration^38^. We confirmed that the antiferromagnetic configuration of Fe atoms is established in the defective cells after the structural optimizations under no constraint regarding the total magnetic moment, even though the initial spin state around *V*_O_^••^ is set to the ferromagnetic order.

## Data Availability

The data that support the findings of this study are available from the corresponding authors on reasonable request.
